# The complete mitochondrial genome of *Fannia canicularis* (Diptera: Fanniidae)

**DOI:** 10.1080/23802359.2022.2134744

**Published:** 2022-10-26

**Authors:** Mihong Ge, Dehuan Wang, Huan Liang, Juhong Zhu, Xianfeng Shi, Junhua Tian

**Affiliations:** a Wuhan Academy of Agricultural Sciences, Wuhan, China; b Wuhan Center for Disease Control and Prevention, Wuhan, China

**Keywords:** Mitogenome, Fanniidae, *Fannia canicularis*

## Abstract

*Fannia canicularis* (Linnaeus, 1761) is a species from the family Fanniidae. In this study, we sequenced and analyzed the complete mitochondrial genome of *F. canicularis* for the first time. The circular mitogenome is 15,826 bp in length, and includes 13 protein-coding genes (PCGs), 22 transfer RNA genes, two ribosomal RNA genes, and a non-coding control region. The family Fanniidae formed a monophyletic clade in the phylogenetic tree based on 13 concatenated PCGs, sister to three other families in Diptera.

*Fannia canicularis* (little house fly) is a species of fly in the family Fanniidae; it was first named *Musca canicularis* by Linnaeus in 1761 and was included in the genus *Fannia* by Robineau-Desvoidy in 1830. The *Fannia* *canicularis* group was established by Henning in 1955, who reported four canicularis subgroups, including 47 known species (Cheng et al. 2008). The larvae of some species are known vectors of a variety of infectious diseases in animals. Some are agricultural pests that parasitize crops, whereas others can spread pollen and become beneficial insects in agriculture. Although the mitochondrial genome sequences of Diptera have rapidly increased recently (Yang et al. 2011; Palevich et al. 2021), complete mitogenomes of the genus *Fannia* have rarely been reported (Xie et al. 2021). In this study, we sequenced and annotated the complete mitogenome of *F. canicularis* for the first time, which provides evidence for exploring the evolutionary history of these fast-evolving insects from the perspective of the mitochondrial genome (Yan et al. 2021).

Specimens of *F. canicularis* were collected at the Wuhu Agricultural Ecological Park (30°42′35″ N, 114°28′11″ E) in Huangpi District, Wuhan City, Hubei Province, China, and were identified at species level using taxonomic keys of Lu (1982). The experimental procedures were approved by the Ethics Committee of Wuhan Center for Disease Control and Prevention (reference number: WHCDCIRB-K-2021017). The voucher specimen was deposited in 95% ethanol at −70 °C at the Wuhan Academy of Agricultural Sciences (http://www.wuhanagri.com/, Mihong Ge, gemihong@wuhanagri.com) under voucher number 2020082701. Genomic DNA was extracted using the Mollusc DNA Kit D3373 (OMEGA, USA), according to the manufacturer’s recommendations. The library was prepared and sequenced on an Illumina NovaSeq 6000 platform at Novogene (Tianjin, China) for paired end 150 bp sequencing. The mitogenome was assembled with MEGAHIT v1.2.6, and annotated using MITOS WebServer (Bernt et al. 2013).

The complete mitogenome of *F. canicularis* is 15,826 bp in length (GenBank number OK623536) with a nucleotide composition of 39.58% A, 38.65% T, 9.13% G, and 12.43% C (AT content 78.23%). It includes 37 genes with 13 protein-coding genes (PCGs), two ribosomal RNAs (rRNAs), 22 transfer RNAs (tRNAs), and one non-coding control region. This is the second mitogenome in the family Fanniidae, which was reported to contain a control region (Ren et al. 2020). Among the mitogenome elements, nine PCGs and 14 tRNAs are encoded by the H-strand, and the remaining four PCGs, eight tRNAs, and two rRNAs are encoded by the L-strand. In addition, *nad2*, *atp8*, *nad3*, *nad5*, and *nad6* used ATT as start codons, *cox2*, *atp6*, *cox3*, *nad4*, *nad4l*, and *cytb* used ATG, and *cox1* used TCG, which is commonly observed in other available Diptera mitogenomes (Li et al. 2016; Ge et al. 2019). There are four types of termination codons: T (*cox2*), TA (*nad5*, *nad4*), TAG (*cytb*), and TAA (*nad2*, *cox1*, *atp8*, *atp6*, *cox3*, *nad3*, *nad4*L, *nad6*, and *nad1*). Furthermore, there were 11 intergenic spacers (76 bp in total) ranging from 1 to 18 bp and 16 overlapping regions (32 bp in total) ranging from 1 to 15 bp.

To clarify the phylogenetic position of *F. canicularis*, we constructed a phylogenetic tree of 27 species from the order Diptera based on the concatenated sequences of the 13 PCGs. *Chlorops oryzae* from the family Chloropidae was used as the outgroup (Figure 1). In the phylogenetic tree, *F. canicularis* was present within the family Fanniidae, forming a cluster with *Fannia spp*. Since only two limited mitogenomes of *Fannia spp.* are available in GenBank, the mitochondrial genome of *F. canicularis* we report will contribute to the molecular identification and evolutionary research of flies.

**Figure 1.**
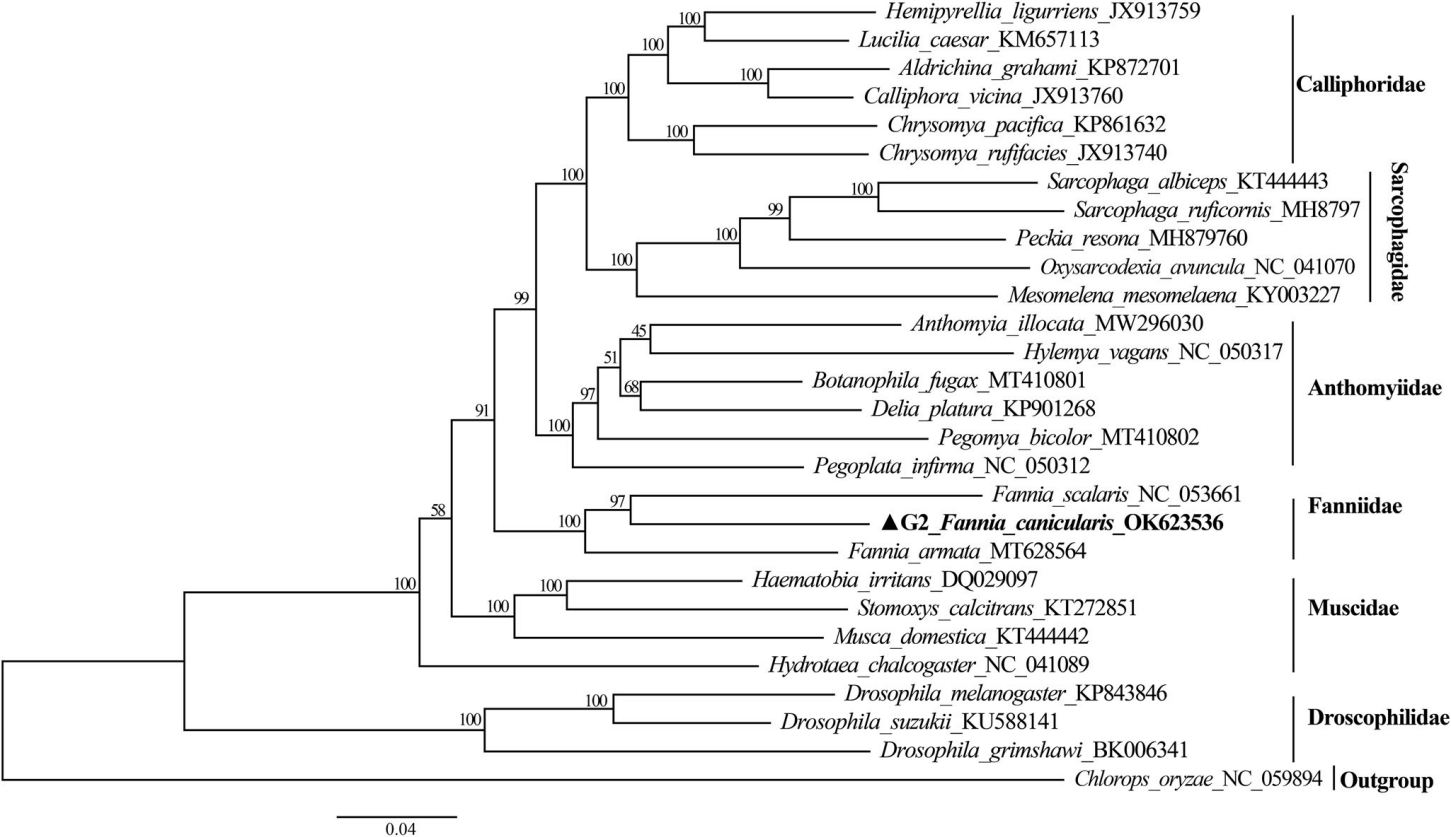
Phylogenetic analysis of *F. canicularis* based on 13 protein-coding genes using the maximum-likelihood (ML) method with iqtree 1.6.12.

## Author contributions

This paper was conceived and designed by Junhua Tian, drafted by Mihong Ge and revised critically for intellectual content by Xianfeng Shi. Analysis of the data was conducted by Dehuan Wang, Huan Liang, and Juhong Zhu. Junhua Tian was involved in the final approval of the version to be published. All authors agree to be accountable for all aspects of the work.

## Data Availability

The genome sequence data that support the findings of this study are openly available in GenBank of NCBI (https://www.ncbi.nlm.nih.gov/nuccore/OK623536) under the accession no. OK623536. The associated BioProject, SRA, and Bio-Sample numbers are PRJNA778448, SRR16842736, and SAMN22960271, respectively.
